# Longitudinal Covid-19 effects on child mental health: vulnerability and age dependent trajectories

**DOI:** 10.1186/s13034-023-00652-5

**Published:** 2023-09-04

**Authors:** Linda Larsen, Stefan Kilian Schauber, Tonje Holt, Maren Sand Helland

**Affiliations:** 1 Division of Mental & Physical Health, Norwegian Institute of Public Health, P.O. Box 222, Skøyen, 0213 Oslo, Norway; 2 Faculty of Medicine, University of Oslo, P.O. Box 1078, 0316 Blindern, Norway

**Keywords:** Covid-19, Anxiety and depression, Externalizing difficulties, Children and adolescents, Vulnerability, Longitudinal study

## Abstract

**Background:**

Few longitudinal studies have investigated the extended long-term impact of the Covid-19 pandemic for children’s and adolescents’ mental health, and a lack of uniform findings suggest heterogeneity in the impact of the pandemic.

**Methods:**

This study investigated child and adolescent mental health symptoms across four occasions (pre-pandemic, initial lockdown, second lockdown, and society post reopening) using data from the Dynamics of Family Conflict study. Child and adolescent depressive vulnerability, age, and sex were explored as trajectory moderators. Children and adolescents (*N* = 381, *M*_age_ = 13.65, *SD* = 1.74) self-reported their anxiety, depression, and externalizing symptoms. Mixed effects analyses were performed to investigate trajectories across measurement occasions and interaction terms between occasion and moderator variables were included to better understand the heterogeneity in the impact of the pandemic.

**Results:**

Children and adolescents reported increases in anxiety symptoms at the second lockdown (*t*(523) = −3.66, *p* < .01) and when society had reopened (*t*(522) = −4.90, *p* < .001). An increase in depression symptoms was seen when society had reopened relative to the three previous measurement occasions (*p*s < 0.01). Depressive vulnerability moderated the trajectory for anxiety symptoms (*F*(3,498) = 3.05, *p* = .028), while age moderated the trajectory for depression symptoms (*F*(3,532) = 2.97, *p* = .031).

**Conclusion:**

The delayed and negative impact on children’s and adolescents’ mental health underscores the need for continued monitoring, and implementation of support systems to help and mitigate further deterioration.

**Supplementary Information:**

The online version contains supplementary material available at 10.1186/s13034-023-00652-5.

More than three years on from when the World Health Organization (WHO) declared the coronavirus (Covid-19) a global pandemic [1] concerns remain about the pandemic’s impact on the wellbeing of children and adolescents [2]. So far findings indicate a large heterogeneity in children’s reactions to the pandemic, including reports of both negative and positive experiences (e.g., increased connectedness with friends and family, respite from daily stressors) [3, 4]. Due to the scarcity of longitudinal research that go beyond the earlier phases of the pandemic, include pre-pandemic measures, and address moderating influences of child mental health vulnerability, age, and sex, it is difficult to ascertain the long-term impact of the pandemic on children and adolescents [5–7]. The present study addresses these gaps by examining child and adolescent mental health during a 19-month period, and by investigating how the longitudinal effects of the pandemic vary with depressive vulnerability and child age and sex. The study is unique in spanning a period from three months before the onset of the nationwide lockdown in Norway, until the lockdown was lifted and replaced by localized social distancing protocols. It thus covers a period before the pandemic as an important baseline measure, the two national lockdowns and a period when society was reopening. Two other unique aspects are that child depressive vulnerability was obtained well before the pandemic onset and is therefore not confounded by the pandemic, and that the study uses child self-report rather than parent-report.

## Implications of the pandemic on child and adolescent internalizing and externalizing symptoms

Several systematic reviews and meta-analyses on the consequences of the pandemic for child and adolescent mental health have been published [8], including some focusing exclusively on longitudinal studies [2, 9]. Yet, more longitudinal investigations that use child self-reports are needed, as parental reports may have inherent problems such as underreporting, especially for child internalising difficulties [10]. Results from longitudinal studies are mixed. For example, using data from 12 different longitudinal studies, Barendse et al. [11] found a significant increase in adolescents’ depressive symptoms but not anxiety symptoms from before the pandemic to six months after. Similar results were found in a UK birth cohort study [12]. However, others have found evidence of increases in adolescents’ mental health problems for symptoms of both anxiety and depression [7, 13, 14], although, at least in the Norwegian study by Hafstad et al. [14] the effect was driven by an increase in adolescents’ age suggesting only a negligeable effect on adolescents’ mental health symptoms.

While age and sex effects on child and adolescent mental health are common, in the context of the Covid-19 pandemic results are discrepant. For example, von Soest et al. [15] found that depressive symptoms increased significantly more among girls than boys, and more among younger than older adolescents. This in part contrasts a large-scale study in Iceland that found depressive symptoms increased more among older than younger adolescents [16]. Another study similarly showed larger symptom increases among adolescent girls than boys from before the pandemic to two months after the initial social distancing protocols, but failed to find a moderating effect of age [7]. In terms of pre-existing child vulnerability, a robust Dutch study with adolescents assessed before the pandemic (i.e., baseline), and then again early (April 2020) and later (January 2021) in the pandemic, found that, counter to expectation, adolescents in the *clinical* or *borderline range* of emotional and behavioural problems at baseline experienced decreased symptoms of anxiety and depression during the early pandemic, and that the decrease was substantially larger among adolescents in the *clinical range*. Later in the pandemic, symptom levels returned to baseline levels [5].

Other research have focused on children’s externalizing difficulties, especially using parental report on the Strength and Difficulties Questionnaire [17]. One study by Feinberg et al. [18] found that parents were 2.5 times more likely to report clinical levels of externalizing difficulties among 8-10-year-olds during the first months of the pandemic compared to before the pandemic. Similarly, Ravens-Sieberer et al. [19] found a significant increase in the proportion of children (7–17 years) who scored in the *abnormal range* for conduct problems and hyperactivity in the early phase of the pandemic. On the other hand, Achterberg et al. [20] found a small downward trend, albeit non-significant, for externalizing difficulties between 2019 and the first Covid-19 lockdown among 10-13-year-olds. Two explanations are offered for this finding; one that the pandemic has buffered children against daily stressor (e.g., at school) and increased parent-child interactions [4], and the other that the lockdown has decelerated the normative developmental decrease in externalizing behaviours [21]. Few studies have used child self-reported externalizing difficulties. One exception, however, is a large Norwegian study of 11-19-year-olds that found that externalizing difficulties remained stable across the two major lockdowns in Norway (i.e., April and December 2020). Unfortunate the study did not include pre-pandemic data [22]. There is also limited research on potential moderation effects, and the few existing studies have resulted in mixed findings. One study found no effect of sex on hyperactivity-inattention [18], while another found an increase in externalizing difficulties for girls only [23]. Moreover, at least one study show that adolescents with high pre-existing vulnerability (i.e., being in the highest quartile on externalizing difficulties pre-Covid) have decreased externalizing difficulties over time, although this did not reach statistical significance, relative to adolescents without vulnerability whose symptom level remained stable or increased only slightly [23].

## The need for high-quality longitudinal studies focusing on pre-existing vulnerability

In view of the heterogeneity of these findings, the importance of investigating child and adolescent mental health from before and at multiple occasions during the Covid-19 pandemic is underscored. In addition, it is important to consider various developmental aspects that may affect findings in longitudinal studies of children and adolescents. One such aspect is an *accumulation of risk* (e.g., school closure, isolation from friends) during the prolonged Covid-19 pandemic that may deplete the physiological stress response system in children. Furthermore, the pandemic incurs changes to the processes that help promote *resilience* in children such as self-regulation. These risk and protective factors have implications for how children respond to and cope with for example, home confinement and other stressors related to government or local social distancing protocols. Another aspect is the *sleeper effect*, which denotes delayed effects contrasted with immediate effects after an event. In the context of the Covid-19 pandemic, effects may be seen on child psychological wellbeing that only manifest after some time [6]. The sleeper effect has been extensively studied, for example in relation to parental divorce where young adults who experienced parental divorced in childhood have been shown to have more insecure adult attachments and negative adult relationship outcomes [24, 25]. Finally, the *sensitizing effect* suggests that children who have pre-existing vulnerability may be particularly “sensitive” to the effects of an adverse event or experience such as the Covid-19 pandemic, but the pandemic itself may also be a sensitizing effect. That is, it may lower the threshold for coping with later difficulties or adversity in children without pre-existing vulnerabilities [6]. Longitudinal studies that (1) include pre-Covid-19 measurements of mental health symptom as well as pre-existing vulnerabilities; (2) use the same sample over time; (3) measure symptoms at several timepoints throughout the course of the pandemic and (4) rely on child and adolescent self-report rather than parent-reports, are paramount to generating important knowledge about the implications of the pandemic on children’s lives. With this knowledge, policy makers, health professional and others working with or addressing issues concerning child and adolescent mental health may adequately perform the task of securing children’s future [5, 23].

### The present study

In this study, we investigate the trajectories of children’s and adolescents’ (henceforth, children) anxiety, depression, and externalizing symptoms from three months before the initial lockdown (i.e., March 2020) to 16 months later (i.e., society reopened again). Moreover, we investigate whether these trajectories are moderated by children’s depressive vulnerability assessed between 4 and 24 months prior to the pandemic, age, and sex. Given the paucity of longitudinal studies that go beyond the initial phase of the Covid-19 pandemic and mixed findings in the literature, we do not have specific hypotheses regarding the trajectories of mental health symptoms or the moderating effects of depressive vulnerability, age, and sex. As far as we are aware, our study is one of few studies to investigate the longitudinal impact of the pandemic on child self-reported externalizing difficulties, to have one pre-pandemic and three peri-pandemic assessment points, and to have a psychological vulnerability measure from before our pre-pandemic baseline measure. The novelty of this study therefore allows for a better understanding of the long-term implications of the pandemic and the social distancing protocols on children and addresses an important knowledge gap by investigating moderation effects of different child-related factors.

## Method

### Study design and participants

We used data from the Dynamics of Family Conflict Study (FAM-C), an ongoing longitudinal survey study aimed at increasing knowledge about family dynamics and conflicts in Norwegian families. FAM-C has more than 2800 participating families, recruited through family counselling centres from December 2017 to July 2019, when families attended mandatory mediation (in relation to divorce/relationship dissolution), counselling or family therapy. FAM-C is a multi-informant study with both parents and up to five children from the same family participating. Children 12 years and older complete online questionnaires covering a wide range of topics, while trained interviewers complete structured interviews (comprising the same questions) with younger children (7–11 years).

**Fig. 1:**
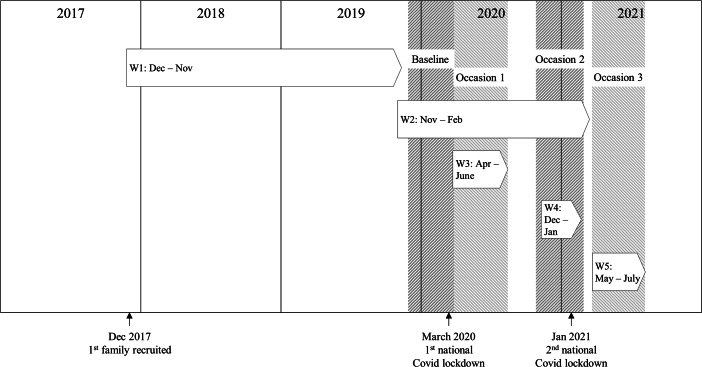
Timeline for the Data Collection Waves (W) in the FAM-C Study

Figure 1 presents a timeline for the data collection waves in the FAM-C study. To address our research aims, the data were organized according to the date that children participated and into the following four measurement occasion: *Baseline* (pre-pandemic; December 10, 2019 – March 12, 2020); *Occasion 1* (first major lockdown; March 12 – June 1, 2020); *Occasion 2* (second lockdown; November 1, 2020 – January 23, 2021) and *Occasion 3* (society reopening; May 20 – July 1, 2021). Detailed information about the FAM-C study including the data collection waves, and information about the Norwegian Government’s response to the Covid-19 pandemic is found in Supplementary Information 1 and 2, available online.

The sample in the present study comprised 381 children with data available on at least one outcome variable on at least one measurement occasion. Children’s mean age at baseline was 13.65 years (*SD* = 1.74, range: 11-17.83). There were 141 children who participated on one occasion, 108 participated on two occasions, 101 participated on three occasions, and 31 participated on all four occasions. Across the different occasions, 107 children (28%) participated at Baseline, 204 (54%) at Occasion 1, 230 (60%) at Occasion 2, and 243 (64%) at Occasion 3. The lower response rate at Baseline is a result in part of the FAM-C study design, where Wave 2 surveys were sent to children (and parents) successively and around 18–24 months after they enrolled in the study. Thus, the W2 data collection from which the Baseline data are drawn, covers a restricted period that naturally reduces the potential sample size. See Table 1 for sample characteristics.

**Table 1:** Sample Demographic Descriptive Statistics by Measurement Occasion (N = 381)

	All children^a^ (n = 381)	Baseline (n = 107)	Occasion 1 (n = 204)	Occasion 2 (n = 230)	Occasion 3 (n = 243)
Age, year	13.65 (1.74)	14.16 (1.70)	13.96 (1.79)	14.67 (1.77)	15.15 (1.74)
Sex					
Male	157 (41.21)	38 (35.51)	74 (36.27)	82 (35.65)	93 (37.27)
Female	224 (58.79)	69 (64.49)	130 (63.73)	148 (64.35)	150 (61.73)
Siblings					
Yes	354 (94.40)	104 (97.20)	187 (93.03)	215 (95.56)	234 (96.30)
No	21 (5.60)	3 (2.80)	14 (6.97)	10 (4.44)	9 (3.70)
Parents cohabiting					
Yes	108(28.72)	25 (23.36)	61 (30.50)	63 (27.75)	69 (28.40)
No	268 (71.28)	82 (76.64)	139 (69.50)	164 (72.25)	174 (71.60)

*Note*. ^a^Based on children’s age at baseline, that is, December 2019, irrespective of whether they participated at Baseline or not

### Outcomes

*Symptoms of anxiety* were assessed with three items the Screen for Child Anxiety Related Disorders (SCARED) [26]. Items are rated on a 3-point scale with the responses “not true or hardly ever true” (0), “somewhat true or sometimes true” [1] and “very true or often true” [2]. *Symptoms of depression* were assessed with seven items from the Mood and Feelings Questionnaire (MFQ) [27]. Items are rated on a 3-point scale with the responses “not true” (0), “sometimes” [1] and “true” [2]. Both scales have demonstrated good psychometric properties [26–28] and in the present study, internal reliability across the different occasions was between α = 0.56 (Occasion 1) and α = 0.67 (Occasion 2 and 3) for SCARED and between α = 0.84 (Baseline) and α = 0.88 (Occasion 3) for MFQ. *Externalizing difficulties* were assessed with six items from the Strength and Difficulties Questionnaire (SDQ) [29]. Items are rated on a 3-point scale with responses “not true” (0), “somewhat true” [1] and “certainly true” [2]. The externalising difficulties subscale from SDQ has previously demonstrated good internal reliability [30], in the present study reliability was between α = 0.60 (Occasion 1) and α = 0.70 (Occasion 2). The mental health variables were assessed on all four measurement occasions and average scores were used in the analyses.

### Other variables

Occasion (0 = Baseline; 1 = Occasion 1; 2 = Occasion 2; 3 = Occasion 3), age at baseline, sex (0 = female; 1 = male), and pre-existing vulnerability (i.e., self-reported symptoms of depression) were used as predictors. Age at baseline was mean centred on each occasion before being entered into the analyses. Depressive vulnerability at W1, that is, before baseline and around 4–24 months before the onset of the pandemic, was assessed with the MFQ using self-report [27], and mean scores were used in the analyses. Sibling status (0 = no siblings; 1 = has siblings) and parental cohabitation status (0 = cohabit; 1 = not cohabiting) were also included as covariates in the analyses. Missing data on these variables were negligible (less than 2%).

### Statistical analyses

Data analyses were performed in R version 4.2.2 [31] using the lme4 [32], emmeans [33], psych [34], dplyr [35], ggplot2 [36], and ggpubr [37] packages. We employed a series of increasingly complex mixed effects models to investigate the mean change in each outcome measures at each measurement occasion. For each outcome, we first estimated an intercept only model with a random intercept for each participant (Model 1) and we then added a second random intercept for each family, as we had siblings participate (Model 2). To decide on the random effect structure, we compared models using the ANOVA function and by inspecting the variance partitioning in Model 2. Even if Model 2 was not significantly better, we nonetheless proceeded with this model when the variance partitioning between the two random effects was equal or close to equal. This suggests equal or near equal importance of both. Second, we added occasion as a predictor (Model 3). Third, we added the remaining predictors and covariates to the model (Model 4). And finally, we estimated three interaction models: between occasion and W1 depressive symptoms (Model 5), occasion and age (Model 6), and occasion and sex (Model 7). We dichotomized age such that children younger than 13 years were coded 0 and children 13 years or older we coded 1. In Norway, children enter secondary school the year they turn 13, and it thus presents an important time of developmental and maturational change. It can therefore be seen as a critical age, where the pandemic may differentially have affected those below and above 13 years. We used the *emmeans* function to derive estimated marginal means, and significant interaction effects were followed up by post hoc testing. Finally, we performed participant sensitivity analyses.

## Results

**Fig. 2:**
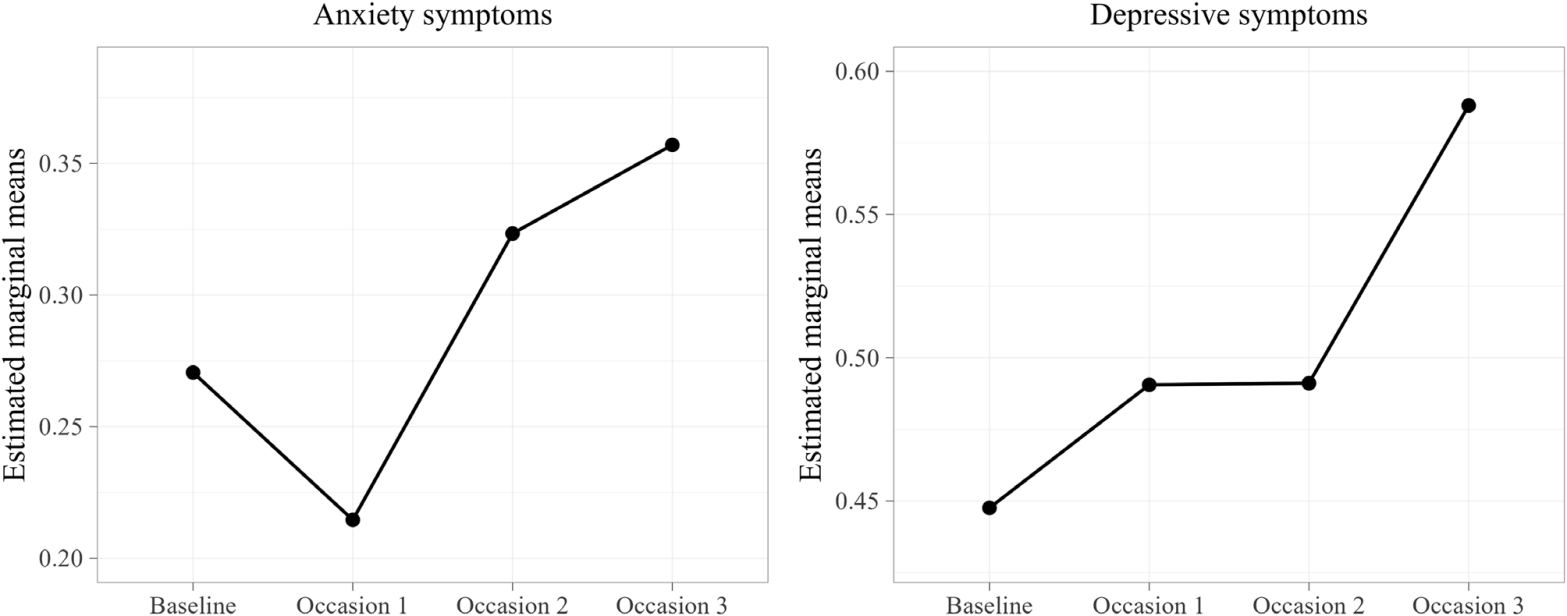
Trajectories for Symptoms of Anxiety and Depression across Baseline (pre-pandemic), Occasion 1 (first major lockdown), Occasion 2 (second lockdown) and Occasion 3 (society reopening)

Initial model comparisons (i.e., Models 1 and 2) showed that random intercepts for subject and family should be used for modelling all three outcomes. Moreover, results from the main effects analyses (i.e., Model 4) showed a significant occasion main effects for symptoms of anxiety and depression (anxiety: *F*(3,515) = 8.73, *p* < .001; depression: *F*(3,539) = 6.56, *p* < .001), but not for externalizing symptoms. This suggests a change over time across children in internalizing symptoms, but not in externalizing symptom. This effect addresses our first research aim regarding the trajectories of child mental health before and during the pandemic, and we probed this further for internalizing symptoms using pairwise comparisons. Results showed a steady increase in child self-reported symptoms of anxiety between Occasion 1 and Occasion 2 (*t*(523) = −3.66, *p* < .01) and also between Occasion 1 and Occasion 3 (*t*(522) = −4.90, *p* < .001). While there was a decreased between Baseline and Occasion 1, this did not reach statistical significance. For symptoms of depression, we found an increased symptom level at Occasion 3 relative to the other three occasions (Baseline: *t*(566) = −3.63, *p* < .01; Occasion 1: *t*(533) = −3.14, *p* < .01; Occasion 2: *t*(482) = −3.35, *p* < .01), but there were no significant differences between Baseline, Occasion 1 and Occasion 2 (*p*s > 0.05). See Fig. 2; Table 2 for an overview of the results.

**Table 2:** Results from Mixed-Effects Models without Interaction Effects (Model 4)

*Predictors*	Anxiety symptoms			Depressive symptoms			Externalizing symptoms		
	*B*	*CI*	*p*	*B*	*CI*	*p*	*B*	*CI*	*p*
(Intercept)	0.35	0.17–0.53	< 0.001	0.35	0.16–0.53	< 0.001	0.69	0.48–0.89	< 0.001
*Fixed Effects*									
W1 Depressive Symptoms	0.24	0.15–0.33	< 0.001	0.55	0.46–0.65	< 0.001	0.29	0.19–0.39	< 0.001
Siblings (ref = has sibs.)	−0.09	−0.25–0.07	0.284	−0.06	−0.23–0.10	0.449	−0.09	−0.28–0.09	0.327
Parents cohabiting (ref = cohabit.)	−0.01	−0.09–0.07	0.750	0.03	−0.06–0.11	0.546	0.08	−0.01–0.17	0.080
Age at baseline	0.02	−0.00–0.04	0.065	0.01	−0.01–0.04	0.202	−0.03	−0.05 – −0.00	0.035
Sex (ref = male)	−0.26	−0.33 – −0.19	< 0.001	−0.22	−0.30 – −0.15	< 0.001	−0.07	−0.15–0.01	0.091
Occasion 1 (1st lockdown)	−0.06	−0.13–0.02	0.139	0.04	−0.04–0.12	0.284	0.05	−0.03–0.12	0.252
Occasion 2 (2nd lockdown)	0.05	−0.02–0.13	0.152	0.04	−0.03–0.12	0.267	0.03	−0.05–0.10	0.487
Occasion 3 (reopening)	0.09	0.02–0.16	0.017	0.14	0.06–0.22	< 0.001	0.06	−0.02–0.13	0.128
*Random Effects*									
σ^2^	0.08			0.09			0.07		
τ_participants_	0.06			0.06			0.07		
τ_family_	0.02			0.02			0.03		
ICC	0.50			0.48			0.60		
N_participants_	369			372			354		
N_family_	304			307			294		
Observations	764			771			707		
Marginal R^2^ / Conditional R^2^	0.198 / 0.602			0.323 / 0.648			0.098 / 0.640		

**Fig. 3:**
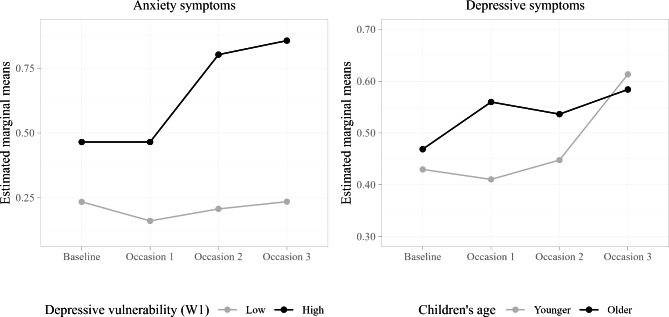
Interaction Effects between Occasion and Child Characteristics across Baseline (pre-pandemic), Occasion 1 (first major lockdown), Occasion 2 (second lockdown) and Occasion 3 (society reopening)

Results from the interaction analyses (Models 5–7, see Supplementary Information 3), addressing the moderation effects of depressive vulnerability, and child age and sex on the trajectories of child mental health, showed a significant interaction effect for depressive vulnerability on the trajectory of anxiety symptoms (*F*(3,498) = 3.05, *p* = .028). When we explored this further using the minimum (i.e., 0) and maximum (i.e., 2) values for depressive vulnerability, we found that the minimum group (low vulnerability) had a stable trajectory across the entire study-period, and they generally (except at Baseline) had significantly lower scores than the maximum group (high vulnerability). The trajectory for the high vulnerability group, however, increased significantly between Baseline and Occasion 3 (*t*(535) = −2.74, *p* = .032) and between Occasion 1 and Occasion 2 and Occasion 3, respectively (*t*(503) = −2.91, *p* = .020 and *t*(490) = −3.41, *p* < .01), suggesting a worsening of self-reported anxiety symptom level across time for more vulnerable children (see Fig. 3, left panel).

The only other significant moderation effect was for age on the trajectory of depression symptoms (*F*(3,532) = 2.97, *p* = .031). Bearing in mind that we dichotomized age in the interaction analyses, the result suggests that the symptom level for depression for older children remained stable across the study-period (*p*s > 0.05). For younger children, however, it was stable across Baseline, Occasion 1 and Occasion 2 (*p*s > 0.05), but there was an increasing trajectory between Occasion 2 and Occasion 3 (*t*(473) = −3.72, *p* < .01; see Fig. 3, right panel). The symptom level was significantly increased for older children relative to younger children at Occasion 1 only (*t*(710) = −2.69, *p* < .01). See Table 3 for estimated marginal means.

**Table 3:** Summary of Estimated Marginal Means (Standard Error) by Moderator Variable and Mental Health Outcome

	Anxiety symptoms					Depressive symptoms					Externalizing difficulties			
	Baseline	Occasion 1	Occasion 2	Occasion 3		Baseline	Occasion 1	Occasion 2	Occasion 3		Baseline	Occasion 1	Occasion 2	Occasion 3
Depressive vulnerability														
Low	0.22 (0.06)	0.15 (0.05)	0.20 (0.05)	0.22 (0.05)		0.29 (0.07)	0.25 (0.06)	0.24 (0.06)	0.35 (0.06)		0.67 (0.07)	0.68 (0.06)	0.67 (0.06)	0.70 (0.06)
High	0.45 (0.14)	0.45 (0.11)	0.79 (0.11)	0.85 (0.11)		1.05 (0.15)	1.40 (0.12)	1.43 (0.11)	1.50 (0.11)		1.14 (0.15)	1.32 (0.12)	1.25 (0.11)	1.29 (0.11)
Child age														
Younger	0.21 (0.07)	0.21 (0.05)	0.32 (0.05)	0.29 (0.05)		0.43 (0.07)	0.41 (0.05)	0.45 (0.06)	0.61 (0.06)		0.80 (0.08)	0.80 (0.06)	0.86 (0.06)	0.88 (0.06)
Older	0.29 (0.06)	0.21 (0.05)	0.32 (0.05)	0.39 (0.05)		0.47 (0.06)	0.56 (0.05)	0.54 (0.05)	0.58 (0.05)		0.77 (0.06)	0.82 (0.06)	0.77 (0.06)	0.81 (0.06)
Child sex														
Female	0.40 (0.06)	0.32 (0.05)	0.46 (0.05)	0.49 (0.05)		0.55 (0.06)	0.61 (0.05)	0.60 (0.05)	0.71 (0.05)		0.79 (0.07)	0.86 (0.06)	0.84 (0.06)	0.86 (0.06)
Male	0.12 (0.07)	0.13 (0.05)	0.17 (0.06)	0.20 (0.05)		0.35 (0.07)	0.38 (0.06)	0.38 (0.06)	0.47 (0.06)		0.77 (0.08)	0.76 (0.06)	0.75 (0.06)	0.81 (0.06)

### Sensitivity analyses

Sensitivity analyses were performed to gauge potential differences between participants that participated on one occasion (*n* = 141) and participants that participated on more than one occasion (*n* = 240). Generally, the groups were similar, except for sex, where the ratio was almost equal among participants that participated on one occasion (48% vs. 52%) but favoured females among participants that participated on more than one occasion (67% vs. 33%). A chi-square test of independence between sex and group was significant (*X*^2^(1, N = 2) = 11.02, *p* < .001).

## Discussion

The present study adds to the scarce knowledgebase on the long-term implications of the Covid-19 pandemic on children by investigating the trajectories of children’s mental health during an extended period, and by exploring child-specific trajectory moderators including depressive vulnerability, age, and sex. Our findings show delayed effects on mental health which underscores the importance of extended longitudinal studies. These findings may also help policy makers in their efforts to develop preparedness plans for future unprecedented events, and they may guide mental health professionals and school personnel to support children now and in the future.

Contrasting some previous findings of increases in symptoms of anxiety and depression early in the pandemic [7, 13], we found an increasing trajectory in internalizing symptoms later in the pandemic. A possible explanation for the contrasting findings might be found in the cultural context, where Norway has one of the strongest social and economic safety nets in the world. It also appears that very liberal cut-offs for anxiety and depression were used in the study by Chen et al. [13], meaning that the probability of falling above cut-off at follow-up was much higher.

In our study, we found that symptoms of anxiety and depression manifested at different times. Specifically, for symptoms of anxiety, the increase arose around the second lockdown in Norway (i.e., Occasion 2) and then remained stable through mid-2021 (i.e., Occasion 3). It is maybe surprising that the initial lockdown in March 2020, did not incur a spike in anxiety symptoms, and in fact, our data showed a small, albeit non-significant, decrease from Baseline to the initial lockdown (i.e., Occasion 1). As others before us have suggested, the initial lockdown may have provided children with some degree of respite from daily stressor that in turn countered the fears and worries associated with the pandemic [4]. We have previously found evidence for this, where children reported to have fewer emotional reactions, but a similar level of worry reactions, when asked during the initial lockdown to retrospectively compare how they were feeling [38].

It appears that only when the second major lockdown was enforced, this reflected in children’s symptoms of anxiety increasing. Perhaps the constant information stream about the pandemic (i.e., including hospitalization, infection, and casualty rates), together with the novelty of home-schooling “wearing off”, and prolonged lack of physical contact with friends and peers, may have exacerbated children’s feelings of uncertainty and worry about the future. Interestingly though, the trajectory for depressive symptoms only increased once the social distancing protocols were gradually removed and society reopening. This delayed increase, or *sleeper effect*, underscores the importance of the long-term perspective on the consequences of the pandemic on children’s lives [5]. Another possibility is that the increase in symptoms was related to the reopening of society. It is likely that this period would have been associated with some stress and difficulty in re-establishing daily routines, and that the transition back to school for some children may have been associated with dread or difficulty responding to and/or handling in-person school expectations. The reopening may also have been difficult for the parents causing a ripple effect on the family and the children. Although this is purely speculative, such a hypothesis could be tested by exploring the relationship between parents’ reactions to the reopening (e.g., stress levels, wellbeing, work-family life balance) and their child’s mental wellbeing, during the pandemic including when society was reopening. While the delayed increase in the trajectory for depressive symptoms relative to the trajectory for anxiety symptoms is interesting, it is not entirely surprising, as this developmental sequential relationship is commonly observed [39], although some evidence points to a reciprocal rather than sequential relationship between the two [40]. Intuitively, if children have been excessively anxious and concerned about the pandemic over an extended period and experienced the pandemic as particularly taxing, this could lead to increased symptoms of depression as a kind of “wear and tear effect” that fits with the sequential notion of anxiety preceding depression.

Finally, we found that child externalizing difficulties remained stable over time. This may reflect a negative impact of the pandemic long-term, if subscribing to the idea of a decreasing developmental trajectory of externalizing difficulties in children [20]. However, it is difficult to say if our self-report measure simply was not good enough at detecting a difference, as there are strengths in using both self- and parent-report to assessing externalizing difficulties.

A pertinent question is to what extent, if any, child mental health in the context of Covid-19 vary with child characteristics. This information is essential for policy makers and health-professionals to develop targeted preparedness and contingency plans. Somewhat surprising and in contrast to previous studies [7, 15, 23], our results suggest that boys and girls shared similar mental health trajectories. However, we did find an interesting moderation effect of child age on depression later in the pandemic, namely, between the second lockdown (i.e., Occasion 2) and when society was returning to normal (i.e., Occasion 3). While there was a small, but non-significant, increase for older children, the increase for younger children was much larger. This suggests that even if younger children thought the lockdown periods were difficult, the reopening potentially presented even more difficulty. This was not true for older children, something that fits with the developmental perspective that younger children will thrive better with more time with their family and parents, which in turn may have buffered them against some of the negative effects of the pandemic and increased their sense of security in the uncertainty. Therefore, the reopening of society and returning to “normal” with less time at home with family may have been particularly difficult for the younger children. Alternatively, in view of younger children generally having lower levels of depressive symptoms than older children (i.e., at Baseline, Occasion 1, and Occasion 2), it is possible that the deterioration was related to this difference among younger and older children.

For the moderating effect of depressive vulnerability on the trajectory of anxiety symptoms, our results seem to support a partial *sensitizing* and *sleeper effect* [6]. Children with low vulnerability had a stable trajectory throughout the study-period and children with high vulnerability generally exhibited more anxiety symptoms and furthermore, showed a marked increase over time. This emerged at the second lockdown and then remained stable. This seems to support a proposition that children had a delayed understanding and appreciation of the gravity of the pandemic and its consequences for the future only after the first lockdown. It seems that children who were already experiencing some mental health difficulty, were more negatively impacted by the pandemic.

It is too early to conclude regarding the lasting consequences of the pandemic for children. Nonetheless, our findings suggest that continued and extra support for younger children is needed. While the Norwegian government’s tactic to shield younger children from the social distancing protocols (e.g., more time at school and less home-schooling than older children) may have had some merit, not enough focus was placed on supporting them back into an opening society again. It is not too late to boost the support system in schools for children. It ought to be a focus point in the event of another unprecedented event, for example through more school health nurses (or psychologists) in schools or mental wellbeing being incorporated into school systems for example using the WHO’s Mental Health in Schools guide [41]. Our findings also suggest that children with pre-existing vulnerability were more negatively impacted, and thus, a future aim should be to ensure that vulnerable children are identified and provided adequate support both during and after a lockdown. It may be that for these children (early) school closure inadvertently had a greater negative impacted than the pandemic itself (e.g., viral infection) would have had. In the event of another pandemic, policy makers should consider equally the risks and consequences of infection and school closure/national lockdown on children drawing on the expertise of those working with children, something the Norwegian government and Directorate of Health has been criticized for not doing [42].

This study has some strengths that deserve mentioning. Parents may underreport on their child’s internalizing and externalizing difficulties [43] and thus, it is a strength of the present study that we were able to use child self-report on measures of anxiety and depression, as well as externalizing difficulties, which few Covid-19 studies have. Another strength is the inclusion of four measurement occasions – one pre-pandemic and three during the long-term of the pandemic. Moreover, given the richness and length of the FAM-C study, we were in a unique position to include a measure of depressive vulnerability from several months prior to the onset of the pandemic (i.e., before Baseline), both as a control variable and as a moderator of children’s mental health trajectories. The study also has some limitations that deserve mentioning. Children as young as 7 years participate in the FAM-C study, but we were for practical reasons prevented from using data from children younger than 11 years (at Baseline). They did not participate through the usual structured interview at W4 and W5 and would therefore not have been able to contribute to the longitudinal aspect of the present study. Another limitation is the lack of detailed information about the level of social distancing protocols during the study period for individual children. Restriction levels may have been experienced differently by the children in the study, especially as these were stricter and endured longer in the larger cities, something that may have impacted the findings. Finally, caution should be exerted in generalizing the findings from the present study, as children were from families recruited when in contact with family counselling centres across Norway. Although this is a low-threshold service, it could be argued that families are more vulnerable (i.e., help-seeking families, going through parental relationship dissolution or conflict). Taken together with the large proportion of children in our sample whose parents are separated/divorced, differences exist relative to the general population. But rightly, it is important to investigate this sample, as there are heightened concerns that children in vulnerable families may have been disproportionally affected by the pandemic.

In conclusion, this study highlights the importance of continued efforts to assess the impact of the pandemic on child mental health with a focus on the long-term effects even after society has returned to a kind of new normal. We have added to the scarce knowledgebase about child characteristics as moderators of the trajectories of child mental health in the Covid-19 context. However, it is important to build on these findings and we encourage researchers to particularly address child psychological vulnerability and different socioeconomic factors (e.g., household density or number of children in the home, family ethic background, parental occupation, and income) as trajectory moderators. Particularly the socioeconomic moderators would help to shed light on why the pandemic has impacted some children (and parents) more than others.

### Electronic supplementary material

Below is the link to the electronic supplementary material.

Supplementary Material 1

Supplementary Material 2

Supplementary Material 3

## Data Availability

The data used in this article can be made available upon request to the first author. To gain access, those requesting access will need to sign a data access agreement.

## Code Availability

The R code can be made available upon request to the first author.
