# Supplementary material for: Longitudinal Covid-19 effects on child mental health: vulnerability and age dependent trajectories
Source: Child Adolesc Psychiatry Ment Health. 2023 Sep 4;17:104. doi: 10.1186/s13034-023-00652-5 (PMC10476387; doi:10.1186/s13034-023-00652-5)
Supplement: Supplementary file 1 — Supplementary Material 1 [file 13034_2023_652_MOESM1_ESM.docx]

**Article title:**

**Longitudinal Covid-19 Effects on Child Mental Health: Vulnerability and Age Dependent Trajectories**

**Author information:**

Linda Larsen^1^, ORCID: 0000-0002-6910-4946

Stefan Kilian Schauber^2^, ORCID: 0000-0002-1832-2732

Tonje Holt^1^, ORCID: 0000-0002-9057-4010

Maren Sand Helland^1^, ORCID: 0000-0001-9728-4094

^1^ Division of Mental & Physical Health, Norwegian Institute of Public Health, P.O. Box 222 Skøyen, 0213 Oslo, Norway

^2^ Faculty of Medicine, University of Oslo, P.O. Box 1078 Blindern 0316, Norway

**Corresponding author:**

Linda Larsen

Mobile: +47 93964849

Email: linda.larsen@fhi.no

**Supplementary 1**

**Data collection waves in the FAM-C study**

Families (i.e., children and their parents) completed the Wave 1 (W1) survey shortly after they were recruited for the study. Approximately 18-24 months later, they were invited to participate in W2, but shortly after W2 was initiated Norway had its first national Covid-19 lockdown and W2 was paused momentarily. An extraordinary data collection (W3) was initiated to assess the impact of the lockdown on the families in the study, and thus, families who had *already* participated in W2 were invited to participate in W3. Families due to participate in W2 *before* August 2020, were invited to complete W2 and W3 at the same time, while families due to participate in W2 *after* August 2020, were invited to participate in W3. The staggering in the W2 administration was implemented for practical reasons and a desire to keep the duration between W1 and W2 as similar as possible across participants. W4 (only children 12 years or older and their parents) and W5 (only children 11 years or older and their parents) were added to track how families coped and progressed through the pandemic long-term.
