# Supplementary material for: Longitudinal Covid-19 effects on child mental health: vulnerability and age dependent trajectories
Source: Child Adolesc Psychiatry Ment Health. 2023 Sep 4;17:104. doi: 10.1186/s13034-023-00652-5 (PMC10476387; doi:10.1186/s13034-023-00652-5)
Supplement: Supplementary file 2 — Supplementary Material 2 [file 13034_2023_652_MOESM2_ESM.docx]

**Article title:**

**Longitudinal Covid-19 Effects on Child Mental Health: Vulnerability and Age Dependent Trajectories**

**Author information:**

Linda Larsen^1^, ORCID: 0000-0002-6910-4946

Stefan Kilian Schauber^2^, ORCID: 0000-0002-1832-2732

Tonje Holt^1^, ORCID: 0000-0002-9057-4010

Maren Sand Helland^1^, ORCID: 0000-0001-9728-4094

^1^ Division of Mental & Physical Health, Norwegian Institute of Public Health, P.O. Box 222 Skøyen, 0213 Oslo, Norway

^2^ Faculty of Medicine, University of Oslo, P.O. Box 1078 Blindern 0316, Norway

**Corresponding author:**

Linda Larsen

Mobile: +47 93964849

Email: linda.larsen@fhi.no

**Supplementary 2**

**Norway in a Covid-19 context for school-aged children**

In Norway, the government closed all schools on March 12, 2020, and children commenced digital home schooling. Schools reopened gradually from April 27 and younger children (up to Grade 4) were the first to return to school. From Mai 11, the school reopening continued, with Grades 5-10 returning to school, granted the schools can adequately follow the requirements for infection control. Norwegian schools were on average closed because of the pandemic for nine weeks, while other OECD countries on average had school closures of 14 weeks.^1^ On May 29, the government introduced a “traffic light” system to reflect the level of restrictions applied by the health authorities to schools in different municipalities and regions across Norway. For the rest of the year including the beginning of the new school year in August, the restriction level was either yellow or red, but there was some variation between primary and secondary schools. On August 1, sports activities for children including competitions were again allowed and children were exempt from the 1-meter distance rule during such events. By November, Norway was experiencing second infection wave, and the government urged everyone to stay at home and to reduce social contact with others. To get the infection rate under control, the government in January 2021, enforces red restriction level for secondary schools and college, which was shortly after reduced to yellow restriction level. Schools in areas with high infection rates were however, to remain on red restriction level. By March, the restriction level in schools and daycares was at yellow restriction level, and two months later this was eased to green restriction level, which means that students are mostly back to in-class teaching. Schools were informed to plan for the new school year starting in August at green restriction level.^2^

The government implemented a four-stage reopening strategy that stresses the need for the infection levels to remain stable before the country can move on to the next level of reduced restrictions. By mid-2021, the country was on stage three, and the government continued the re-opening with more people allowed to visit others at home, less use of home office, and children and youth were allowed more free time activities with less social distancing restrictions. In December, the government reintroduced the traffic light model in schools and daycares and enforced stricter measures to reduce the spread of the Covid-19 disease as case numbers were again increasing. The restrictions were relatively short-lived and on February 12, 2022, the government removed all the remaining social distancing protocols.^2^

Although the population of Norway is only around 5.5 million, it is a vast country with most of the population living in or around the capital Oslo or one or two other larger cities. It is therefore not surprising that there has been much variation in infection and hospitalization rates between municipalities in Norway during the pandemic. We have tried as best as possible to outline above, the social distancing protocols and restriction levels that children had to live with under the pandemic but cannot discount that some children and youths had to live with more severe restriction levels or restrictions levels of longer duration than others. There will always be some variation depending on where in Norway children live. However, families and children in the FAM-C study were recruited from all over Norway and our data therefore reflect the impact of the Covid-19 situation on families and children relative to how different regions of the country were affected.

1. Norske skoler var stengt på grunn av korona i ni uker - mot 14 uker i gjennomsnitt i OECD-landene [Press release]. 8 September 2020, 2020. <https://www.regjeringen.no/no/aktuelt/norske-skoler-var-stengt-pa-grunn-av-korona-i-ni-uker--mot-14-uker-i-gjennomsnitt-i-oecd-landene/id2740614/>

2. Regjeringen.no. Tidslinje: myndighetenes håndtering av koronasituasjonen. Accessed 10.05.2023, <https://www.regjeringen.no/no/tema/Koronasituasjonen/tidslinje-koronaviruset/id2692402/>
