# Supplementary material for: Longitudinal Covid-19 effects on child mental health: vulnerability and age dependent trajectories
Source: Child Adolesc Psychiatry Ment Health. 2023 Sep 4;17:104. doi: 10.1186/s13034-023-00652-5 (PMC10476387; doi:10.1186/s13034-023-00652-5)
Supplement: Supplementary file 3 — Supplementary Material 3 [file 13034_2023_652_MOESM3_ESM.docx]

**Article title:**

**Longitudinal Covid-19 Effects on Child Mental Health: Vulnerability and Age Dependent Trajectories**

**Author information:**

Linda Larsen^1^, ORCID: 0000-0002-6910-4946

Stefan Kilian Schauber^2^, ORCID: 0000-0002-1832-2732

Tonje Holt^1^, ORCID: 0000-0002-9057-4010

Maren Sand Helland^1^, ORCID: 0000-0001-9728-4094

^1^ Division of Mental & Physical Health, Norwegian Institute of Public Health, P.O. Box 222 Skøyen, 0213 Oslo, Norway

^2^ Faculty of Medicine, University of Oslo, P.O. Box 1078 Blindern 0316, Norway

**Corresponding author:**

Linda Larsen

Mobile: +47 93964849

Email: linda.larsen@fhi.no

**Supplementary 3**

*Results from Mixed-Effects Models with W1 Depressive Symptoms by Occasion Interaction Effect (Model 5)*

| *Predictors* | Anxiety symptoms | | | Depressive symptoms | | | Externalizing symptoms | | |
| --- | --- | --- | --- | --- | --- | --- | --- | --- | --- |
|  | *Est* | *CI* | *p* | *Est* | *CI* | *p* | *Est* | *CI* | *p* |
| (Intercept) | 0.4 | 0.21 – 0.59 | **<0.001** | 0.42 | 0.23 – 0.62 | **<0.001** | 0.71 | 0.50 – 0.93 | **<0.001** |
| *Fixed Effects* |  |  |  |  |  |  |  |  |  |
| W1 Depressive Symptoms | 0.12 | -0.05 – 0.28 | 0.166 | 0.38 | 0.21 – 0.55 | **<0.001** | 0.24 | 0.06 – 0.41 | **0.007** |
| Siblings (ref = has sibs.) | -0.08 | -0.24 – 0.08 | 0.329 | -0.06 | -0.23 – 0.10 | 0.455 | -0.09 | -0.28 – 0.09 | 0.322 |
| Parents cohabiting (ref = cohabit.) | -0.01 | -0.09 – 0.07 | 0.817 | 0.03 | -0.05 – 0.11 | 0.494 | 0.08 | -0.01 – 0.17 | 0.077 |
| Age at baseline | 0.02 | -0.00 – 0.04 | 0.077 | 0.01 | -0.01 – 0.04 | 0.221 | -0.03 | -0.05 – -0.00 | **0.037** |
| Sex (ref = male) | -0.26 | -0.33 – -0.19 | **<0.001** | -0.22 | -0.30 – -0.15 | **<0.001** | -0.07 | -0.15 – 0.01 | 0.093 |
| Occasion 1 (1^st^ lockdown) | -0.07 | -0.18 – 0.03 | 0.175 | -0.04 | -0.15 – 0.07 | 0.481 | 0.01 | -0.10 – 0.12 | 0.885 |
| Occasion 2 (2^nd^ lockdown) | -0.03 | -0.13 – 0.08 | 0.607 | -0.05 | -0.16 – 0.06 | 0.383 | 0.00 | -0.10 – 0.11 | 0.94 |
| Occasion 3 (reopening) | 0.00 | -0.10 – 0.10 | 0.992 | 0.06 | -0.05 – 0.17 | 0.312 | 0.03 | -0.07 – 0.14 | 0.560 |
| W1 Dep Symp X Occasion 1 | 0.04 | -0.14 – 0.22 | 0.687 | 0.19 | 0.00 – 0.39 | **0.046** | 0.08 | -0.10 – 0.27 | 0.366 |
| W1 Dep Symp X Occasion 2 | 0.18 | 0.01 – 0.36 | **0.040** | 0.21 | 0.03 – 0.40 | **0.023** | 0.05 | -0.13 – 0.23 | 0.572 |
| W1 Dep Symp X Occasion 3 | 0.20 | 0.02 – 0.37 | **0.027** | 0.19 | 0.01 – 0.38 | **0.041** | 0.06 | -0.12 – 0.23 | 0.514 |
| *Random Effects* | | | | | | | | | |
| σ^2^ | 0.07 | | | 0.09 | | | 0.07 | | |
| τ_participants_ | 0.06 | | | 0.06 | | | 0.07 | | |
| τ_family_ | 0.01 | | | 0.02 | | | 0.03 | | |
| ICC | 0.51 | | | 0.48 | | | 0.6 | | |
| N_participants_ | 369 | | | 372 | | | 354 | | |
| N_family_ | 304 | | | 307 | | | 294 | | |
| Observations | 764 | | | 771 | | | 707 | | |
| Marginal R^2^ / Conditional R^2^ | 0.204 / 0.606 | | | 0.325 / 0.651 | | | 0.098 / 0.640 | | |

*Results from Mixed-Effects Models with Age by Occasion Interaction Effect (Model 6)*

| *Predictors* | Anxiety symptoms | | | Depressive symptoms | | | Externalizing symptoms | | |
| --- | --- | --- | --- | --- | --- | --- | --- | --- | --- |
|  | *Est* | *CI* | *p* | *Est* | *CI* | *p* | *Est* | *CI* | *p* |
| (Intercept) | 0.28 | 0.08 – 0.47 | **0.006** | 0.33 | 0.13 – 0.54 | **0.002** | 0.73 | 0.50 – 0.96 | **<0.001** |
| *Fixed Effects* |  |  |  |  |  |  |  |  |  |
| W1 Depressive Symptoms | 0.25 | 0.16 – 0.34 | **<0.001** | 0.56 | 0.46 – 0.65 | **<0.001** | 0.28 | 0.18 – 0.38 | **<0.001** |
| Siblings (ref = has sibs.) | -0.08 | -0.24 – 0.08 | 0.348 | -0.08 | -0.24 – 0.09 | 0.365 | -0.11 | -0.30 – 0.08 | 0.258 |
| Parents cohabiting (ref = cohabit.) | -0.01 | -0.09 – 0.07 | 0.742 | 0.03 | -0.05 – 0.11 | 0.488 | 0.08 | -0.01 – 0.17 | 0.101 |
| Age (ref = < 13 years) | 0.08 | -0.06 – 0.22 | 0.250 | 0.04 | -0.11 – 0.19 | 0.605 | -0.03 | -0.19 – 0.13 | 0.725 |
| Sex (ref = male) | -0.26 | -0.33 – -0.19 | **<0.001** | -0.22 | -0.30 – -0.14 | **<0.001** | -0.07 | -0.15 – 0.01 | 0.086 |
| Occasion 1 (1^st^ lockdown) | 0.00 | -0.12 – 0.13 | 0.950 | -0.02 | -0.15 – 0.11 | 0.778 | 0.00 | -0.15 – 0.16 | 0.971 |
| Occasion 2 (2^nd^ lockdown) | 0.11 | -0.01 – 0.24 | 0.067 | 0.02 | -0.11 – 0.15 | 0.784 | 0.06 | -0.08 – 0.20 | 0.394 |
| Occasion 3 (reopening) | 0.08 | -0.04 – 0.20 | 0.175 | 0.18 | 0.06 – 0.31 | **0.005** | 0.08 | -0.06 – 0.22 | 0.271 |
| Age X Occasion 1 | -0.08 | -0.24 – 0.07 | 0.288 | 0.11 | -0.05 – 0.28 | 0.189 | 0.05 | -0.13 – 0.23 | 0.595 |
| Age X Occasion 2 | -0.09 | -0.24 – 0.06 | 0.255 | 0.05 | -0.11 – 0.21 | 0.544 | -0.06 | -0.23 – 0.10 | 0.455 |
| Age X Occasion 3 | 0.02 | -0.13 – 0.17 | 0.779 | -0.07 | -0.23 – 0.09 | 0.396 | -0.04 | -0.21 – 0.12 | 0.622 |
| *Random Effects* | | | | | | | | | |
| σ^2^ | 0.07 | | | 0.09 | | | 0.07 | | |
| τ_participants_ | 0.06 | | | 0.06 | | | 0.07 | | |
| τ_family_ | 0.02 | | | 0.02 | | | 0.03 | | |
| ICC | 0.51 | | | 0.48 | | | 0.6 | | |
| N_participants_ | 369 | | | 372 | | | 354 | | |
| N_family_ | 304 | | | 307 | | | 294 | | |
| Observations | 764 | | | 771 | | | 707 | | |
| Marginal R^2^ / Conditional R^2^ | 0.197 / 0.606 | | | 0.327 / 0.653 | | | 0.095 / 0.642 | | |

*Results from Mixed-Effects Models with Sex by Occasion Interaction Effect (Model 7)*

| *Predictors* | Anxiety symptoms | | | Depressive symptoms | | | Externalizing symptoms | | |
| --- | --- | --- | --- | --- | --- | --- | --- | --- | --- |
|  | *Est* | *CI* | *p* | *Est* | *CI* | *p* | *Est* | *CI* | *p* |
| (Intercept) | 0.35 | 0.17 – 0.53 | **<0.001** | 0.34 | 0.15 – 0.53 | **<0.001** | 0.68 | 0.47 – 0.89 | **<0.001** |
| *Fixed Effects* |  |  |  |  |  |  |  |  |  |
| W1 Depressive Symptoms | 0.24 | 0.15 – 0.33 | **<0.001** | 0.55 | 0.46 – 0.65 | **<0.001** | 0.29 | 0.19 – 0.39 | **<0.001** |
| Siblings (ref = has sibs.) | -0.08 | -0.24 – 0.08 | 0.346 | -0.06 | -0.23 – 0.10 | 0.449 | -0.10 | -0.29 – 0.09 | 0.297 |
| Parents cohabiting (ref = cohabit.) | -0.02 | -0.10 – 0.07 | 0.714 | 0.03 | -0.06 – 0.11 | 0.537 | 0.08 | -0.01 – 0.17 | 0.071 |
| Age at baseline | 0.02 | -0.00 – 0.04 | 0.063 | 0.01 | -0.01 – 0.04 | 0.201 | -0.03 | -0.05 – -0.00 | **0.035** |
| Sex (ref = male) | -0.28 | -0.42 – -0.15 | **<0.001** | -0.20 | -0.35 – -0.05 | **0.007** | -0.02 | -0.17 – 0.12 | 0.755 |
| Occasion 1 (1^st^ lockdown) | -0.09 | -0.18 – 0.00 | 0.057 | 0.05 | -0.04 – 0.15 | 0.288 | 0.07 | -0.02 – 0.17 | 0.134 |
| Occasion 2 (2^nd^ lockdown) | 0.05 | -0.04 – 0.14 | 0.249 | 0.05 | -0.05 – 0.14 | 0.315 | 0.05 | -0.04 – 0.14 | 0.292 |
| Occasion 3 (reopening) | 0.09 | -0.00 – 0.18 | 0.051 | 0.15 | 0.06 – 0.25 | **0.001** | 0.07 | -0.03 – 0.16 | 0.157 |
| Sex X Occasion 1 | 0.10 | -0.06 – 0.25 | 0.228 | -0.03 | -0.20 – 0.14 | 0.739 | -0.08 | -0.25 – 0.08 | 0.323 |
| Sex X Occasion 2 | 0.00 | -0.15 – 0.15 | 0.979 | -0.02 | -0.18 – 0.15 | 0.849 | -0.07 | -0.22 – 0.09 | 0.398 |
| Sex X Occasion 3 | 0.00 | -0.15 – 0.15 | 0.973 | -0.04 | -0.20 – 0.12 | 0.663 | -0.02 | -0.18 – 0.13 | 0.773 |
| *Random Effects* | | | | | | | | | |
| σ^2^ | 0.07 | | | 0.09 | | | 0.07 | | |
| τ_participants_ | 0.06 | | | 0.06 | | | 0.07 | | |
| τ_family_ | 0.01 | | | 0.02 | | | 0.03 | | |
| ICC | 0.51 | | | 0.48 | | | 0.6 | | |
| N_participants_ | 369 | | | 372 | | | 354 | | |
| N_family_ | 304 | | | 307 | | | 294 | | |
| Observations | 764 | | | 771 | | | 707 | | |
| Marginal R^2^ / Conditional R^2^ | 0.200 / 0.605 | | | 0.323 / 0.646 | | | 0.099 / 0.638 | | |
